# SELF-ADMINISTRATED ELDER ABUSE INTERVENTION FOR OLDER ADULTS WITH COGNITIVE IMPAIRMENT

**DOI:** 10.1093/geroni/igac059.1673

**Published:** 2022-12-20

**Authors:** Fuad Abujarad, Chelsea Edwards, Brent Vander Wyk, Laura Mosqueda, Ula Hwang, Judith Neugroschl, Richard Marottoli

**Affiliations:** Yale University, Orange, Connecticut, United States; Yale University, New Haven, Connecticut, United States; Yale University, New Haven, Connecticut, United States; University of Southern California, Alhambra, California, United States; Yale University, New Haven, Connecticut, United States; Mount Sinai, New York, New York, United States; Yale University, New Haven, Connecticut, United States

## Abstract

Many elder abuse interventions and tools designed to screen for abuse exclude older adults with cognitive impairments (CI) due to the challenges associated with screening and whether the older adult with CI can reliably report elder abuse. However, it has been shown that older adults with CI are among the most vulnerable to experiencing elder abuse. VOICES is an innovative, automated tablet-based elder abuse screening and prevention intervention that is self-administered by the older adult in the provider’s waiting room or office. The VOICES Elder Abuse Intervention (EAI) provides screening, educational modules, and brief psychoeducational intervention to enhance and improve identification of elder abuse when there are no visible signs of abuse. The VOICES EAI was already proven successful in terms of feasibility and acceptability in cognitively intact older adults in a busy emergency department setting with (N=1,002). In this study we tested the VOICES EAI with (N=30) participants 60 and above with cognitive impairment at a geriatric center using the Montreal Cognitive Assessment (MoCA) to determine cognitive capacity. Experts in the field of geriatrics and cognitive impairment assisted in grouping participants within three cognitive categories: Mild cognitive impairment (MoCA 23-25), mild dementia (MoCA 16-22) or moderate dementia (MoCA 8-15). Of the (N=30) participants, 29 were able to successfully use the VOICES EAI independently, and most participants were satisfied with the tool. We will discuss the findings of this preliminary study and the implications for future research with older adults with CI.

